# Coffee consumption patterns and sleep quality among Saudi Youth: A cross-sectional study

**DOI:** 10.1016/j.sleepx.2026.100187

**Published:** 2026-05-05

**Authors:** Abdulaziz Alkaabba, Ghaiath Hussein, Yazeed Alotaibi, Abdullah Alghasham, Ammar Algezlan, Abdullah Almugren, Abdulaziz Alfadhel, Meshal Alhajrass, Khalid Alwadie, Mohammed Alsubaie, Mohammad Alabdulkareem, Faisal Alaswad, Mosfer A. Al-Walah, Meteb M. Alotiabi

**Affiliations:** a Medical Education and Bioethics, Imam Muhammad Ibn Saud Islamic University (IMSIU), Riyadh, Saudi Arabia; b Discipline of Medical Education, School of Medicine, Trinity College Dublin, Ireland; c College of Medicine, Imam Muhammad Ibn Saud Islamic University (IMSIU), Riyadh, Saudi Arabia; d Department of Physical Therapy, College of Applied Medical Sciences, Taif University, Taif, Saudi Arabia

**Keywords:** Coffee consumption, Sleep quality, Caffeine timing, Saudi Arabia, Circadian rhythms

## Abstract

**Objectives:**

To examine coffee consumption patterns and their associations with sleep quality indicators among Saudi youth, and to identify independent predictors of poor sleep outcomes using multivariate regression.

**Methods:**

A cross-sectional study was conducted among 1458 consenting Saudi youth (aged 16–30 years) using an online questionnaire adapted from the Pittsburgh Sleep Quality Index (PSQI). Chi-square tests examined associations between coffee consumption patterns (type, timing, pre-bedtime cessation) and sleep variables. Binary logistic regression identified independent predictors of late bedtime (sleeping after midnight), adjusting for demographic covariates.

**Results:**

The sample was predominantly female (69.4%, n = 1012) and aged 18–22 years (65.7%, n = 958). Evening/night-time coffee consumption was a strong independent predictor of sleeping after midnight (OR = 3.04, 95% CI: 2.30–4.02, p < 0.001), adjusting for cessation strategy, sex, age, and student status. Chi-square analyses confirmed that coffee consumption timing showed highly significant associations with bedtime patterns (χ^2^ = 57.806, p < 0.001), sleep duration (χ^2^ = 42.759, p < 0.001), and daytime sleep behaviours (χ^2^ = 27.247, p < 0.001). Pre-bedtime cessation timing was significantly associated with bedtime patterns (χ^2^ = 73.075, p < 0.001) and nighttime awakenings (χ^2^ = 28.912, p = 0.007).

**Conclusions:**

Temporal aspects of coffee consumption demonstrate stronger and more consistent associations with sleep quality than coffee type selection. Evening coffee use is an independent risk factor for delayed sleep timing. Strategic timing interventions may be more effective than content-based restrictions for improving sleep health in Saudi youth.

## Highlights

• Evening/night-time coffee consumption independently predicted sleeping after midnight (OR = 3.04, 95% CI: 2.30–4.02).
• Temporal aspects of coffee consumption showed stronger associations with sleep outcomes than coffee type.
• Among those sleeping after midnight, 84.6% reported evening or night-time coffee use.
• Age and gender were significant demographic moderators of sleep quality and cessation behaviours.
• Timing-based interventions may be more effective than content-based restrictions in this population.

## 1 Introduction

Coffee consumption has become deeply integrated into the daily routines of young adults worldwide, driven by academic pressures, social interactions, and cultural practices [1,2]. In Saudi Arabia, this pattern is particularly pronounced given the country's vibrant café culture and the traditional role of coffee in hospitality and social gatherings [3,4].

Caffeine, the primary psychoactive compound in coffee, exerts its effects by antagonising adenosine receptors in the brain, disrupting the natural sleep–wake cycle [5,6]. it serves multiple functions beyond alertness enhancement, including social bonding, ritual participation, and stress management [5,7].

This mechanism leads to delayed sleep onset, reduced sleep efficiency, and altered sleep architecture, particularly when consumption occurs within 6 h of bedtime. **[** [7,8] The half-life of caffeine ranges from three to 7 h in healthy adults, with considerable individual variability in metabolism [9,10].

Recent research from the Middle East and North Africa (MENA) region has highlighted concerning levels of caffeine consumption among university students, often linked to academic stress and irregular sleep schedules [11,12].

In Saudi Arabia specifically, studies have documented high rates of coffee consumption among young adults, with limited awareness of caffeine's impact on sleep quality [13,14].

This knowledge gap is particularly concerning given the cultural significance of coffee in Saudi society.

The relationship between caffeine consumption and sleep disturbances is moderated by several factors, including timing of consumption, individual sensitivity, demographic characteristics, and awareness of caffeine's effects [8].

Previous research has suggested that younger individuals may be more susceptible to caffeine's sleep-disrupting effects, while gender differences in caffeine metabolism and sensitivity have also been documented [[15], [16], [17]].

However, comprehensive multivariate studies examining these relationships within culturally specific contexts remain limited.

This study addresses this gap by: (1) examining associations between coffee consumption patterns and sleep quality indicators in a large Saudi youth sample using chi-square analyses; and (2) extending prior descriptive work by identifying independent predictors of late bedtime using binary logistic regression, controlling for key demographic covariates.

## 2 Methods

### 2.1 Study design and participants

A cross-sectional, observational study was conducted among Saudi youth aged 16–30 years using an online self-administered questionnaire. Stratified random sampling ensured representation across different regions of Saudi Arabia, with recruitment conducted through university portals, student organisations, and social media platforms between January and April 2024. Of 1488 total responses, 1458 participants who provided explicit informed consent were included in all analyses.

### 2.2 Data collection instrument

The questionnaire was developed based on the Pittsburgh Sleep Quality Index (PSQI) framework [18] and supplemented with caffeine-specific variables capturing cultural preferences and consumption patterns. Five sections were included: demographic information, coffee consumption patterns (frequency, timing, quantity, type, environment, reasons for consumption), sleep quality assessments, awareness of coffee–sleep relationships, and strategic behaviours related to caffeine cessation.

To assess the psychometric properties of the questionnaire, internal consistency reliability was evaluated using Cronbach's alpha for the core sleep quality items adapted from the PSQI framework and the supplementary caffeine-specific items. The PSQI-derived items (covering sleep duration, sleep disturbance, sleep efficiency, sleep quality, sleep latency, daytime dysfunction, and use of sleep medication) demonstrated acceptable to good internal consistency (Cronbach's α = 0.76). The caffeine-specific items added to capture cultural consumption patterns and cessation behaviours demonstrated acceptable internal consistency (Cronbach's α = 0.71). Item-total correlations were examined and all items showed correlations above the 0.30 threshold, indicating adequate contribution to scale reliability. Content validity was established through expert review by two sleep medicine specialists and one cultural consultant familiar with Saudi coffee practices, who confirmed that items adequately represented the domains of interest. A pilot test (n = 30) confirmed item clarity and face validity prior to full deployment.

### 2.3 Statistical analysis

Data analysis was performed using SPSS version 28.0. Chi-square tests examined associations between categorical variables (significance threshold p < 0.05; degrees of freedom [df] and chi-square values [χ^2^] reported). Demographic factors (age, gender, employment status, region) were analysed as potential moderating variables.

Binary logistic regression was performed to identify independent predictors of late bedtime (defined as habitual sleep onset after midnight), which emerged as the sleep outcome most strongly associated with coffee timing in bivariate analyses. Predictor variables were: evening/night-time coffee consumption (any use after 18:00 versus none), cessation strategy (no strategic cessation versus any timing strategy), gender, age (ordinal: 0 = under 18 through 4 = over 30), and student status. Results are presented as odds ratios (OR) with 95% confidence intervals (CI). Nagelkerke R^2^ is reported as a measure of model fit.

### 2.4 Ethical considerations and use of artificial intelligence

This study was conducted in accordance with the guidelines of the Declaration of Helsinki. All procedures were approved by the Institutional Review Board of Imam Muhammad Ibn Saud Islamic University (Ethics approval: IMISIU-IRB 25/686). Written informed consent was obtained digitally from all participants prior to questionnaire completion, with assurance of anonymity and the right to withdraw at any time.

Artificial intelligence tools (Microsoft Copilot and Grammarly) were used strictly for language editing, grammar correction, and formatting assistance. No AI tools were employed for generating, analysing, or interpreting scientific data. All findings, interpretations, and conclusions represent the original intellectual contribution of the authors.

## 3 Results

### 3.1 Participant demographics

A total of 1458 consenting Saudi youth participated (Table 1). The sample was predominantly female (69.4%, n = 1012), with the majority aged 18–22 years (65.7%, n = 958). Most participants were Saudi nationals (95.0%), students (76.7%, n = 1143), and had monthly incomes below 5000 SAR (81.5%). Geographic distribution favoured the Central Region (33.4%) and Western Region (29.3%).

**Table 1:** Demographic characteristics of study participants (N = 1458).

Characteristic	Category	n (%)
Gender	Female	1012 (69.4%)
	Male	446 (30.6%)
Age group	Under 18 years	188 (12.9%)
	18–22 years	958 (65.7%)
	23–26 years	160 (11.0%)
	27–30 years	57 (3.9%)
	Over 30 years	95 (6.5%)
Region	Central	487 (33.4%)
	Western	427 (29.3%)
	Eastern	245 (16.8%)
Employment status	Student	1143 (76.7%)
	Full-time employee	167 (11.5%)
	Unemployed	141 (9.7%)

### 3.2 Coffee consumption patterns and motivations

The analysis revealed highly significant associations between motivations for coffee consumption and behavioural patterns. The relationship between drinking motivations and coffee types consumed demonstrated the strongest association (χ^2^ = 935.046, df = 288, p < 0.001), indicating strategic selection of specific coffee varieties based on intended outcomes. Consumption timing showed an equally robust association with motivations (χ^2^ = 261.695, df = 96, p < 0.001), and consumption locations varied significantly by motivation (χ^2^ = 456.675, df = 225, p < 0.001) (Table 3).

Gender demonstrated strong associations with consumption locations (χ^2^ = 82.980, df = 9, p < 0.001), with females more likely to consume coffee at home (46.2%) compared with males (14.9%). Age showed significant associations with consumption locations (χ^2^ = 88.215, df = 36, p < 0.001). Employment status significantly influenced consumption motivations (χ^2^ = 887.957, df = 576, p < 0.001) and locations (χ^2^ = 273.228, df = 162, p < 0.001) (Table 2).

**Table 2:** Coffee consumption patterns by demographic characteristics.

Variable	Category	At Home	At Work/School	In Cafés	χ^2^ (p)
Gender	Female	687 (46.2%)	134 (9.0%)	144 (9.7%)	82.980 (<0.001)
	Male	221 (14.9%)	102 (6.9%)	74 (5.0%)	
Employment	Student	732 (49.2%)	129 (8.7%)	174 (11.7%)	273.228 (<0.001)
	Full-time employee	52 (3.5%)	73 (4.9%)	19 (1.3%)

**Table 3:** Coffee consumption motivations and behavioural associations.

Association	χ^2^ Value	df	p-value
Motivations × Coffee types	935.046	288	<0.001 ***
Motivations × Daily timing	261.695	96	<0.001 ***
Motivations × Consumption locations	456.675	225	<0.001 ***
Motivations × Pre-bedtime cessation	153.974	128	0.059 NS

***p < 0.001; NS = not significant.

### 3.3 Coffee timing and sleep quality – bivariate analyses

Coffee consumption timing demonstrated a highly significant association with bedtime patterns (χ^2^ = 57.806, df = 6, p < 0.001), sleep duration (χ^2^ = 42.759, p < 0.001), and daytime sleep behaviours (χ^2^ = 27.247, p < 0.001). Pre-bedtime coffee cessation timing showed the strongest association with bedtime patterns (χ^2^ = 73.075, df = 8, p < 0.001) and significant associations with nighttime awakenings (χ^2^ = 28.912, p = 0.007). Coffee type was associated with bedtime patterns (χ^2^ = 33.783, p = 0.013) but not with daytime sleep behaviours (χ^2^ = 19.696, p = 0.350). Sleep onset latency was not significantly associated with any coffee consumption variable. Full results are presented in Table 4.

**Table 4:** Sleep quality indicators and coffee consumption pattern associations (χ^2^ values; p-values in parentheses).

Sleep Quality Indicator	Coffee Type	Daily Timing	Pre-bedtime Cessation
Bedtime patterns	33.783 (0.013)*	57.806 (<0.001)***	73.075 (<0.001)***
Sleep duration	23.860 (0.005)**	42.759 (<0.001)***	31.245 (0.001)**
Time to fall asleep	15.984 (0.192) NS	18.756 (0.094) NS	22.134 (0.076) NS
Nighttime awakenings	51.914 (0.003)**	36.754 (<0.001)***	28.912 (0.007)**
Daytime sleep behaviours	19.696 (0.350) NS	27.247 (<0.001)***	39.552 (<0.001)***
Sleep aid usage	12.300 (0.197) NS	4.014 (0.260) NS	5.460 (0.243) NS
Technology use before bed	25.383 (0.553) NS	6.167 (0.723) NS	12.960 (0.372) NS

*p < 0.05; **p < 0.01; ***p < 0.001; NS = not significant.

### 3.4 Independent predictors of late bedtime – logistic regression

Of the 1458 consenting participants, 55.8% (n = 813) reported habitual sleep onset after midnight. Among those with late bedtime, 84.6% reported evening or night-time coffee use, compared with 64.0% of those with earlier bedtimes.

Binary logistic regression (N = 1458; Table 6) identified evening/night-time coffee consumption as a strong, independent predictor of late bedtime (OR = 3.04, 95% CI: 2.30–4.02, p < 0.001) after adjusting for cessation strategy, gender, age, and student status. Older age was also an independent predictor (OR = 1.36 per ordinal level, 95% CI: 1.16–1.59, p < 0.001). Cessation strategy (no timing versus any timing), female sex, and student status were not independently significant after adjustment. Model fit was acceptable (Nagelkerke R^2^ = 0.497).

**Table 5:** Demographic influences on key coffee and sleep variables (selected).

Variable	Age χ^2^ (p)	Gender χ^2^ (p)	Employment χ^2^ (p)
Coffee consumption locations	88.215 (<0.001)***	82.980 (<0.001)***	273.228 (<0.001)***
Bedtime patterns	51.874 (<0.001)***	12.567 (0.084) NS	43.157 (0.192) NS
Average sleep duration	42.759 (<0.001)***	15.541 (0.001)**	79.461 (0.014)*
Nighttime awakenings	13.995 (0.301) NS	36.754 (<0.001)***	52.891 (0.028)*
Daytime sleep behaviours	29.252 (<0.001)***	14.293 (0.001)**	58.746 (0.010)*
Strategic coffee cessation	57.378 (<0.001)***	26.633 (<0.001)***	130.994 (<0.001)***
Evening coffee effects on sleep	22.845 (0.029)*	22.077 (<0.001)***	67.963 (0.096) NS

*p < 0.05; **p < 0.01; ***p < 0.001; NS = not significant.

**Table 6:** Binary logistic regression: independent predictors of late bedtime (sleeping after midnight), N = 1458.

Predictor	OR	95% CI	p-value	Sig.
Evening/night-time coffee use	3.04	2.30–4.02	<0.001	***
No cessation strategy (vs. any)	1.15	0.84–1.56	0.389	NS
Early cessation ≥3h before bed	0.82	0.58–1.15	0.241	NS
Female sex	1.18	0.93–1.51	0.177	NS
Age (ordinal, each level)	1.36	1.16–1.59	<0.001	***
Student status	0.89	0.64–1.26	0.573	NS

OR = odds ratio; CI = confidence interval. Late bedtime = habitual sleep onset after midnight (n = 813, 55.8%). Evening/night-time coffee = any coffee consumed after 18:00. ***p < 0.001; NS = not significant. Nagelkerke R^2^ = 0.497.

### 3.5 Demographic influences

Age, gender, and employment status emerged as highly influential moderating factors across multiple domains (Table 5). Strategic coffee cessation behaviours showed strong age-related patterning (χ^2^ = 57.378, p < 0.001), with older youth more likely to implement timing strategies. Female participants demonstrated greater sensitivity to evening coffee consumption (χ^2^ = 22.077, p < 0.001) and nighttime awakenings (χ^2^ = 36.754, p < 0.001). Employment status significantly influenced both consumption contexts and sleep duration (Table 7, Table 8).

**Table 7:** Gender differences in sleep quality and coffee cessation.

Variable	Female	Male	χ^2^ (p-value)
Sleep Duration			15.541 (0.001)**
<6 h	156 (10.7%)	55 (3.8%)	
6-7 h	445 (30.5%)	189 (13.0%)	
7-8 h	398 (27.3%)	156 (10.7%)	
>8 h	187 (12.8%)	113 (7.7%)	
Nighttime Awakenings			36.754 (<0.001)***
Difficulty returning to sleep	298 (20.4%)	67 (4.6%)	
Easy to return to sleep	732 (50.2%)	391 (26.8%)	
Strategic Coffee Cessation			26.633 (<0.001)***
Stop 3-6 h before bed	194 (13.3%)	109 (7.5%)	
Avoid evening coffee completely	106 (7.3%)	80 (5.5%)	
No strategic timing	730 (50.1%)	269 (18.4%)	
Evening Coffee Effects			22.077 (<0.001)***
No sleep disruption	234 (16.0%)	141 (9.7%)	
Mild sleep difficulty	412 (28.3%)	170 (11.7%)	
Severe sleep disruption	384 (26.3%)	147 (10.1%)	

**p < 0.01; ***p < 0.001.

**Table 8:** Employment status effects on coffee consumption and sleep.

Variable	Students	Full-time	Part-time	Unemployed	χ^2^ (p-value)
**Primary Coffee Location**					273.228 (<0.001)***
At Home	732 (50.2%)	52 (3.6%)	8 (0.5%)	112 (7.7%)	
At Work/School	129 (8.8%)	73 (5.0%)	7 (0.5%)	6 (0.4%)	
In Cafés	174 (11.9%)	19 (1.3%)	6 (0.4%)	17 (1.2%)	
**Sleep Duration**					79.461 (0.014)*
<6 h	156 (10.7%)	28 (1.9%)	4 (0.3%)	23 (1.6%)	
6-7 h	445 (30.5%)	67 (4.6%)	11 (0.8%)	62 (4.3%)	
7-8 h	398 (27.3%)	52 (3.6%)	6 (0.4%)	41 (2.8%)	
>8 h	144 (9.9%)	20 (1.4%)	3 (0.2%)	15 (1.0%)	
**Strategic Coffee Cessation**					130.994 (<0.001)***
1-2 h before bed	186 (12.8%)	23 (1.6%)	4 (0.3%)	28 (1.9%)	
3-6 h before bed	202 (13.9%)	49 (3.4%)	8 (0.5%)	31 (2.1%)	
Avoid evening coffee	156 (10.7%)	31 (2.1%)	5 (0.3%)	19 (1.3%)	
No strategic timing	599 (41.1%)	64 (4.4%)	7 (0.5%)	7 4.3%)	

*p < 0.05; ***p < 0.001.

## 4 Discussion

### 4.1 Principal findings

This study offers two analytically distinct contributions. First, bivariate analyses confirm a consistent pattern in which temporal aspects of coffee consumption are more strongly associated with sleep quality outcomes than coffee type selection — a finding consistent with published evidence on caffeine pharmacokinetics [7,8]. Second, and more critically, logistic regression establishes that evening/night-time coffee consumption is an independent predictor of sleeping after midnight (OR = 3.04, p < 0.001), even after controlling for cessation awareness, gender, age, and student status.

The finding that 84.6% of late-night sleepers reported evening or night-time coffee use, compared with 64.0% of those who slept earlier, underscores the clinical significance of coffee timing at a population level. Importantly, the absence of a significant effect for cessation strategy after adjustment suggests that it is the direct consumption of coffee in the evening that drives delayed sleep timing, rather than general awareness or intent to stop — a distinction with practical implications for health education messaging.

### 4.2 Temporal primacy in caffeine–sleep relationships

The consistent superiority of timing variables over coffee type in predicting sleep outcomes aligns with caffeine's pharmacokinetics, where the timing of consumption relative to sleep onset determines the degree of adenosine receptor blockade during critical sleep initiation and maintenance periods [7,8]. The highly significant association between pre-bedtime coffee cessation timing and bedtime patterns (χ^2^ = 73.075, p < 0.001) suggests that individuals who implement strategic timing behaviours may be responding to either conscious awareness of caffeine's effects or learned associations between consumption timing and sleep quality. This supports interventions focusing on temporal consumption patterns rather than absolute caffeine avoidance [19,20].

The finding that caffeine timing significantly affected sleep duration and nighttime awakenings but not sleep onset latency (χ^2^ = 34.155, p = 0.162) aligns with polysomnographic evidence that caffeine's adenosine receptor antagonism primarily affects slow-wave sleep and sleep consolidation rather than the initial wake-to-sleep transition [19,20]. This differential effect suggests that caffeine's primary influence is on sleep maintenance rather than sleep initiation — a nuance relevant to clinical counselling.

### 4.3 Demographic moderation effects

The age-related progression in strategic coffee cessation behaviours (χ^2^ = 57.378, p < 0.001) suggests that sleep hygiene awareness develops progressively through late adolescence and early adulthood, consistent with the ongoing maturation of prefrontal cortical systems supporting executive decision-making through the mid-twenties [21,22].

Older youth were more likely to implement timing strategies — a finding with clear implications for age-targeted public health messaging.

The consistent pattern of gender differences across consumption motivations, sleep quality indicators, and strategic behaviours aligns with broader research on gender differences in caffeine metabolism and sleep architecture [16,[23], [24], [25], [26], [27]].

Female participants demonstrated greater sensitivity to evening coffee consumption and more pronounced sleep disruption, consistent with established gender differences in chronotype tendencies and sleep spindle characteristics [19,20].

The logistic regression finding that gender was not independently significant for late bedtime — despite its bivariate associations with multiple sleep variables — suggests that gender's influence on sleep timing is partly mediated through its relationship with evening coffee use patterns.

### 4.4 Cultural and social context

The finding that consumption locations vary significantly with motivations (χ^2^ = 456.675, p < 0.001) highlights the complex social dimensions of coffee consumption in Saudi society. The traditional role of coffee in Saudi hospitality creates unique challenges for implementing timing-based interventions, as social consumption may not always align with optimal sleep hygiene practices. The predominance of home consumption, particularly among females (46.2%), may represent a culturally specific avenue for household-level interventions.

### 4.5 Clinical and public health implications

The logistic regression finding — that evening coffee consumption triples the odds of sleeping after midnight independently of whether participants report a cessation strategy — provides a specific and actionable target for clinical counselling. Healthcare providers should counsel Saudi youth to avoid coffee after 18:00 rather than relying on general cessation awareness, which does not independently reduce late-night sleep timing after adjustment.

The significant employment status effects suggest that workplace-based and institution-based programmes may be particularly effective for reaching specific populations. The different patterns between students and employed individuals indicate that intervention messages should be adapted to address lifestyle contexts and demands [28].

### 4.6 Limitations

The cross-sectional design limits causal inference. The predominantly female sample may limit generalisability to male populations. Self-reported data introduces potential recall bias and social desirability effects. The study did not measure actual caffeine content consumed or individual metabolic differences. The logistic regression outcome (late bedtime) is a proxy for sleep timing rather than a direct measure of sleep quality; future studies should incorporate actigraphy or polysomnography. The cultural specificity of the sample, while informative for Saudi public health, limits generalisation to other populations. A further limitation relates to the binary operationalisation of sleep timing: “late bedtime” was defined as habitual sleep onset after midnight (00:00), which imposes a dichotomy on what is inherently a continuous variable. This approach risks information loss and may marginally overestimate the statistical significance of associations compared with analyses treating sleep timing as continuous or using empirically derived cut-offs. The midnight threshold was selected because it emerged as the most clinically salient boundary in bivariate analyses and has practical communicability for public health messaging; however, future studies should model sleep timing as a continuous outcome (e.g., using linear regression on clock time or circadian phase) or explore data-driven thresholds to verify the robustness of these findings.

## 5 Conclusion

This study provides robust evidence, including a novel logistic regression analysis of raw survey data, that evening and night-time coffee consumption is an independent predictor of delayed sleep timing in Saudi youth (OR = 3.04, 95% CI: 2.30–4.02). Temporal aspects of coffee consumption consistently outperformed coffee type in predicting sleep outcomes across bivariate analyses. These findings support the prioritisation of timing-based clinical counselling — specifically discouraging coffee consumption after 18:00 — over content-based restrictions. Targeted, culturally appropriate interventions accounting for age-specific awareness levels, gender-specific sensitivity patterns, and occupational contexts are needed to improve sleep health in this population.

## CRediT authorship contribution statement

**Abdulaziz Alkaabba:** Writing – review & editing, Writing – original draft, Supervision, Project administration, Methodology, Investigation, Formal analysis, Conceptualization. **Ghaiath Hussein:** Writing – review & editing, Writing – original draft, Supervision, Project administration, Methodology, Formal analysis, Data curation, Conceptualization. **Yazeed Alotaibi:** Writing – review & editing, Writing – original draft, Resources, Investigation, Data curation. **Abdullah Alghasham:** Writing – review & editing, Writing – original draft, Data curation. **Ammar Algezlan:** Writing – review & editing, Writing – original draft, Resources, Methodology, Data curation. **Abdullah Almugren:** Writing – review & editing, Writing – original draft, Data curation, Conceptualization. **Abdulaziz Alfadhel:** Writing – review & editing, Writing – original draft, Resources, Methodology, Data curation. **Meshal Alhajrass:** Writing – review & editing, Writing – original draft, Data curation. **Khalid Alwadie:** Writing – review & editing, Writing – original draft, Data curation. **Mohammed Alsubaie:** Writing – review & editing, Writing – original draft, Data curation, Conceptualization. **Mohammad Alabdulkareem:** Writing – review & editing, Writing – original draft, Resources, Data curation, Conceptualization. **Faisal Alaswad:** Writing – review & editing, Writing – original draft, Validation, Data curation, Conceptualization. **Mosfer A. Al-Walah:** Writing – review & editing, Writing – original draft, Methodology, Investigation, Funding acquisition. **Meteb M. Alotiabi:** Writing – review & editing, Resources, Funding acquisition.

## Declaration of generative AI and AI-assisted technologies in the manuscript preparation process

During the preparation of this work, the authors used Microsoft Copilot/Grammarly to assist with language editing, proofreading, structural refinement, and completeness checks of the manuscript. The AI tool was not used to generate, analyse, or interpret scientific data, nor to produce original scientific content or conclusions.

After using this tool, the authors carefully reviewed, verified, and edited all outputs to ensure accuracy, coherence, and alignment with the study's objectives and findings. The authors take full responsibility for the content of the published article.

## Financial Support

This research received no specific grant from any funding agency, commercial entity, or not-for-profit organization.

## Declaration of competing interest

The authors declare that they have no known competing financial interests or personal relationships that could have appeared to influence the work reported in this paper.
